# Evaluation of therapy management and outcome in Takotsubo syndrome

**DOI:** 10.1186/s12872-017-0661-8

**Published:** 2017-08-17

**Authors:** Nadine Abanador-Kamper, Lars Kamper, Judith Wolfertz, Witali Pomjanski, Anamaria Wolf-Pütz, Melchior Seyfarth

**Affiliations:** 1 Department of Cardiology, HELIOS Medical Center Wuppertal, University Hospital Witten/Herdecke, Arrenberger Str. 20, 42117 Wuppertal, Germany; 2 Center for Clinical Medicine Witten/Herdecke University Faculty of Health, Wuppertal, Germany; 3 Department of Diagnostic and Interventional Radiology, HELIOS Medical Center Wuppertal, University Hospital Witten/Herdecke, Wuppertal, Germany; 4 Department of Cardiology, Augusta Hospital Düsseldorf, Academic Teaching Hospital of the University Faculty of Health, Düsseldorf, Germany

**Keywords:** Takotsubo cardiomyopathy, Acute coronary syndrome, Cardiovascular magnetic resonance imaging, Antiplatelet therapy, Antithrombotic management, Prognosis

## Abstract

**Background:**

To date there is no validated evidence for standardized treatment of patients with Takotsubo syndrome (TTS). Medication therapy after final TTS diagnosis remains unclear. Previous data on patient outcome is ambivalent. Aim of this study was to evaluate medication therapy in TTS and to analyze patient outcome.

**Methods:**

Within an observational retrospective cohort study we analyzed our medical records and included 72 patients with TTS that underwent cardiovascular magnetic resonance imaging (CMR) after a median of 2 days interquartile range (IQR 1–3.5). We investigated medication therapy at discharge. Medication implementation and major adverse clinical events (MACE) were prospectively evaluated after a median follow-up of 24 months (IQR 6–43). Left ventricular function, myocardial oedema and late gadolinium enhancement were analyzed in a CMR follow-up if available.

**Results:**

Antithrombotic therapy was recommended in 69 (96%) patients including different combinations. Antiplatelet monotherapy was prescribed in 28 (39%) patients. Dual antiplatelet therapy was recommended in 29 (40%) patients. Length of therapy duration varied from one to twelve months. Only in one case oral anticoagulation was prescribed due to apical ballooning with a left ventricular ejection fraction <30%. In all other cases oral anticoagulation was recommended due to other indications. ß-adrenoceptor antagonists and ACE inhibitors were recommended in 63 (88%), mineralocorticoid receptor antagonists were prescribed in 31 (43%) patients. After a median of 2 months (IQR 1.3–2.9) left ventricular function significantly recovered (49.1% ± 10.1 vs. 64.1% ± 5.7, *P* < 0.001) and myocardial oedema significantly decreased (13.5 ± 11.3 vs. 0.6% ± 2.4, *P = <0.001*) in the CMR follow-up. The 30-day mortality was 1%. MACE rate after 24 months was 12%.

**Conclusion:**

Although therapy guidelines for TTS currently do not exist, we found that the majority of patients were treated with antithrombotic and heart failure therapy for up to twelve months. Left ventricular function and myocardial oedema recovered rapidly within the first two months. Outcome analysis showed a low bleeding rate and a high short-term survival. Therefore, TTS patients might benefit from antithrombotic and heart failure therapy at least for the first two months.

## Background

Takotsubo syndrome (TTS) is a form of acute mostly reversible heart failure syndrome. It was first described in Japan. Since that several international reports and mostly observational studies have been described [1]. Early distinguishing from acute myocardial infarction is challenging and its final diagnosis remains unclear in the early stage. In the majority of patients coronary angiogram is inconspicuous; however coexistence of coronary artery disease in TTS is described [2, 3]. Cardiac imaging plays a major role in the diagnosis of TTS and reveals important findings on left ventricular dysfunction. Cardiovascular magnetic resonance imaging (CMR) has a high diagnostic value in patients with TTS [4]. The pathophysiology of TTS seems to be complex, yet there is no current proven pathophysiological mechanism. A stress-induced catecholamine release with impact on the cardiovascular system plays a major role [5–7]. Pathophysiological hypotheses include vascular and myocardial mechanisms that lead to the phenotype of TTS. Blood hyperviscosity caused by vascular dysfunction was found to have an impact [8]. However, intracoronary thrombosis or plaque rupture seems not to be present in TTS [9]. Considering existing data on pathophysiology of TTS patients might benefit from therapy of vascular and acute heart failure. To date no evidence exists for medication treatment and treatment duration of these patients, since there is a lack on randomized clinical trials. In particular, there is no evidence on antithrombotic therapy. All patients initially receive dual antiplatelet therapy on the basis of suspected myocardial infarction. However, there is no recommendation of antithrombotic medication after final diagnosis of TTS. Currently, data are limited on how TTS patients are treated with antiplatelet therapy after discharge. Aim of this study was to evaluate pharmacological treatment and therapy recommendations in TTS and to analyze patient outcome.

## Methods

### Study design

**Fig. 1:**
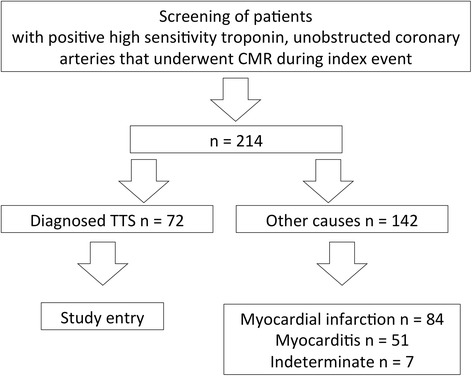
Study flow chart

We retrospectively analyzed the data files of our tertiary care university hospital between 2005 and 2017 for all patients that presented with acute chest pain, elevated cardiac biomarkers, new electrocardiography abnormalities and lack of a target lesion in the angiogram that underwent CMR during the index event (<30 days after admission). Based on the current ESC position statement and the Mayo Clinic diagnostic criteria [10, 11] on TTS diagnosis was established in cases with acute chest pain, transient, acute heart failure with or without apical ballooning or wall motion abnormalities that extended beyond a single epicardial vascular distribution and absence of late gadolinium enhancement (LGE) in CMR. We included 72 patients with TTS (Fig. 1). All patients received standard therapy for acute coronary syndrome according to the current guidelines. All patients <18 years, or with other diagnoses than TTS, a prior history of myocardial infarction, coronary bypass surgery, congenital heart disease and contraindications to CMR and CMR contrast agents were excluded. The local ethics committee approved this study. Patients were contacted directly via telephone and study enrollment was verified. Patients gave written informed consent.

### Laboratory parameters

Blood samples were taken as a part of a standardized routine at the time of hospital admission to measure high-sensitivity troponin T, creatine-kinase myocardial-band (CK-MB) and creatine-kinase (CK)-values daily until normalization. Plasma samples for C-reactive protein (CRP)- and glomerular filtration rate (GFR)-values were evaluated. Peak values of high-sensitivity troponin T, CK-MB, CK, CRP and lowest value of GFR were acquired.

### Medication treatment

Medication at discharge including antiplatelet therapy, anticoagulation, ß-adrenoceptor antagonists (BB), angiotensin converting enzyme receptor inhibitors (ACEi), angiotensin receptor blockers (ARB), mineralocorticoid receptor antagonists (MRA) and their temporal recommendation was evaluated. Implementation of medication was analysed for each patient via standardized questionnaire by telephone contact either with the patient or treating physician.

### CMR protocol and image analysis

All CMR scans were performed on a 1.5-Tesla scanner (Intera Achieva; Philips; Best, Netherlands). CMR studies were independently reviewed by two ESC Level III cardiologists. CMR protocol included analysis of left ventricular function, wall motion analysis, myocardial oedema assessment and LGE. Left ventricular function was assessed by a standard steady-state free precession technique (2D turbo gradient echo sequence) in short, four-chamber, two-chamber and three-chamber views of the left ventricle. As previously validated, T2-weighted black blood turbo-spin-echo sequences (with and without fat saturation pre-pulse) in short axis and in four chamber view covering the complete left ventricle were performed to evaluate myocardial or pericardial oedema [12–16].

LGE images of the left and right ventricles were acquired 10 min after injection of 0,2 mmol/kg of gadoteridol (Prohance®, Bracco-Imaging, Konstanz, Germany). A three-dimensional inversion-recovery turbo gradient echo sequence was used for image acquisition covering the entire left and right ventricle in multiple slices without gap in the short axis, four-, two- and three-chamber long axis views.

CMR parameters were analysed by the use of a specific software-tool (Extended MR Workspace 2.6.3.4, Philips Medical Systems, Best, Netherlands and CMR 42, Version 4.0, Circle Cardiovascular Imaging Inc., Calgary, Canada). Left ventricular ejection fraction (LV-EF) and left ventricular parameters were calculated by assessment of the volumes of the endocardial contours in diastole and systole of the four- and two-chamber slices. Typical wall motion abnormalities were defined as apical, midventricular or basal ballooning of the left ventricle. For the calculation of left ventricular mass endocardial and epicardial contours were drawn manually in the short axis of the left ventricle. Quantitative analysis of myocardial oedema was described as >2SD signal intensity threshold from remote normal myocardium [16]. Myocardial oedema volumes were expressed as percentage of the total LV mass.

LGE was determined by semi-automated quantification in each short axis slice. LGE was defined as hyperenhanced area with signal intensity threshold ≥5 SD above a region of interest of normal “nulled” myocardium [17]. LGE size was expressed as percentage of LV volume given by the sum of the volume of LGE regions for all slices divided by the sum of the total LV myocardial volume.

### CMR follow-up and major adverse clinical events

Patients were screened for CMR follow–up and left ventricular functional parameters were analyzed. Major adverse clinical events (MACE) at follow-up were evaluated via standard questionnaire by telephone contact with the patient or the treating physician after 24 months. Events were verified by hospital charts. MACE was defined as recurrence of TTS or myocardial infarction, stroke or death. The diagnosis of myocardial infarction was based on acute chest pain, pathologically elevated plasma levels for high-sensitivity troponin I, CK-MB and CK, ST-segment elevation of at least 0.1 mV in ≥2 extremity leads or at least 0.2 mV in ≥2 precordial leads and an identifiable target lesion in the angiogram.

### Statistical analysis

Categorical variables are described by frequencies; continuous data are expressed as median with interquartile range or with mean and standard deviation. Wilcoxon matched-pairs signed-ranks test was used to test whether distribution of initial and follow-up CMR parameters differed. We used the Kaplan–Meier method to examine the proportion of patients with events and to estimate the probability of events for different timings. A two-sided *P* value of less than 0.05 was considered to indicate statistical significance. All statistical analysis was performed using STATA/IC 14.2 software (Stat Corp, LP, Texas, USA).

## Results

### Demographic and laboratory findings

Mean age of the patients was 68.8 ± 17.5 years with 67 (93%) female patients. An underlying stress trigger was reported in 36 (50%) patients and prehospital resuscitation in three (4%) patients. In one patient an underlying pheochromocytoma was diagnosed two years after the Takotsubo event. A treated depression was observed in eleven (15%) patients, an epilepsy in one patient. Cardiovascular risk factors included arterial hypertension in 49 (68%) patients, Diabetes mellitus in 7 (10%) patients, current smoking in 9 (12%) patients, hyperlipidemia in 20 (28%) patients and family history for myocardial infarction in 16 (22%) patients. Median body mass index was 24 (IQR 22–29). Median peak value of high-sensitivity troponin T was 371.0 pg/ml (IQR 172–583), of CK-MB 34.0 U/l (IQR 23–47), of CK 188.0 U/l (IQR 137–338) and of CRP 1.20 mg/dl (IQR 0–3). CRP value was obtained for 60 patients (83%). The median lowest GFR-level was 68.0 ml/min/1.73 (IQR 58–80).

### Medication treatment and temporal recommendation

General therapy recommendation of the study population is given in Table 1. All patients received medication therapy. Antithrombotic therapy was recommended in 69 (96%) patients including different combinations of Acetylsalicyl-acid (ASA), P2Y12 antagonists, oral anticoagulation (OAC) and low molecular weight heparin. Out of twelve patients with OAC in one patient the indication was due to new onset of apical ballooning with a left ventricular ejection fraction (LV-EF) <30%. In ten (14%) patients the indication was a history of atrial fibrillation and in one patient a history of deep vein thrombosis. Heart failure medication as BB and ACEi/ARB was recommended in 63 (88%) patients. MRA was prescribed in 31 (43%) patients.

**Table 1:** Recommended therapy management after index event

	Study population *n* = 72
Therapy recommendation	
ASA	59 (81.9%)
Clopidogrel	28 (38.8%)
Prasugrel	2 (2.8%)
Ticagrelor	4 (5.6%)
OAC	12 (16.6%)
LWMH once per day	1 (1.4%)
Statin	47 (65.3%)
Beta-blocker	63 (87.5%)
ACE inhibitor/AT inhibitor	63 (87.5%)
Mineralocorticoid receptor antagonist	31 (43.1%)
Duration of antithrombotic therapy	
No recommendation	41 (56.9%)
With recommendation:	29 (40.3%)
1 month	4 (5.6%)
3 months	8 (11.1%)
6 months	6 (8.3%)
12 months	11 (15.3%)
Reason for OAC	
Atrial fibrillation	10 (13.8%)
Heart failure	1 (1.4%)
Deep vein thrombosis	1 (1.4%)

Data is presented as number of patients and percentage

*LWMH* low weight molecular heparin**,** *OAC* oral anticoagulation

Antithrombotic therapy includes antiplatelet mono- or dual therapy or oral anticoagulation

The different antithrombotic therapy strategies are presented in Table 2. In nine (4%) patients no antiplatelet therapy was prescribed, out of these, six patients had an OAC due to history of atrial fibrillation. An OAC in combination with ASA without temporal recommendation or Clopidogrel for three months was recommended in four (6%) patients.

**Table 2:** Recommended therapy management after index event

	Study population *n* = 72
No antiplatelet therapy	9 (4.2%)
With OAC monotherapy	6
OAC + ASA	3 (4.2%)
Without temporal recommendation	
OAC + Clopidogrel	1 (1.4%)
With temporal recommendation for 3 months	
Monotherapy antiplatelet:	28 (38.9%)
ASA	25 (34.7%)
Without temporal recommendation	24
With temporal recommendation for 3 months	1
Clopidogrel	2 (2.8%)
Without temporal recommendation	1
With temporal recommendation for 6 months	1
Ticagrelor	1 (1.4%)
With temporal recommendation for 12 months	1
Dual antiplatelet therapy:	29 (40.3%)
ASA + Clopidogrel	24 (33.3%)
Without temporal recommendation	5
With temporal recommendation:	19
1 month	2
3 months	4
6 months	4
12 months	8
ASA + Prasugrel	2 (2.8%)
Without temporal recommendation	1
With temporal recommendation for 12 months	1
ASA + Ticagrelor	3 (4.2%)
Without temporal recommendation	0
With temporal recommendation	3
1 month	1
3 months	1
12 months	1
“Triple” therapy with ASA	2 (2.8%)
1 month, Clopidogrel + OAC 6 months, OAC monotherapy	1
1 month, Clopidogrel + OAK 12 months, OAC monotherapy	1

Data is presented as number of patients and percentage

*ASA* Acetylsalicyl Acid, *LWMH* low weight molecular heparin, *OAC* oral anticoagulation

Mono- and dual antiplatelet therapy was the majority of therapy recommendations. In 25 (35%) patients ASA monotherapy was advised, out of these only one patient was given a temporal recommendation for three months. Dual antiplatelet therapy was recommended in 29 (40%) patients with a combination of ASA and Clopidogrel in 24 (33%) patients. In fewer patients dual antiplatelet therapy included the combination of ASA and Clopidogrel or ASA and Ticagrelor, mostly with a temporal recommendation ranging from one to twelve months. Triple therapy was recommended in two patients with a temporal strategy in both patients. Both recommendations of duration differed in length of OAC and Clopidogrel. Duration of antithrombotic therapy was given in 29 (40%) with the longest recommendation for 12 months.

### Implementation of prescribed antithrombotic therapy

Evaluation of prescribed antithrombotic therapy revealed that out of these 29 patients 19 (66%) patients completed the recommended duration. Two patients received a longer treatment due to unawareness of patients or physician. Therapy was aborted earlier in eight (28%) patients secondary to ASA intolerance in three patients and rejection of ASA therapy in three other patients. Gastrointestinal bleeding or malignancy was reported in two patients. The treating physician initiated antiplatelet monotherapy by himself in two patients due to diagnosis of TTS.

### CMR parameters and CMR follow-up

Initial CMR scan was performed after a median of 2 days (IQR1–3.5) during index event in all included patients (Table 3). All patients had a lack of LGE. Mean size of myocardial oedema was measured with 13.5% ± 11.3. In seven (10%) patients a typical wall motion abnormality was not observed in the initial CMR, however, myocardial oedema was significant and typical in all of these patients. CMR follow-up scan was performed in 63 (88%) patients after a median of 2.3 months (IQR 1.3–2.9). Mean LV-EF significantly increased between both scans (49.1% ± 10.1 vs. 64.1% ± 5.7, *P* < 0.001). Furthermore myocardial oedema significantly decreased in the follow-up scan (13.5% ± 11.3 vs. 0.6% ± 2.4, *P* < 0.001).

**Table 3:** Description of differences between CMR parameters of initial CMR and follow-up CMR scan

CMR parameters			
	CMR I *n* = 72	CMR II *n* = 63 (88%)	*p*-value*
LV-EF (%)	49.1 ± 10.1	64.1 ± 5.7	< 0.001
LV-EDVI (ml/m^2^)	76.2 ± 13.7	72.1 ± 13.9	0.198
T2 Volume (%)	13.5 ± 11.3	0.6 ± 2.4	< 0.001
LGE Volume (%)	0.0 ± 0.0	0.0 ± 0.0	
Δ angiogram-CMR (d)	2 (1–3.5)		
Δ CMR I-CMR II (months)	2.3 (1.3–2.9)		

LVEF, LVEDVI, LGE and T2 volumes are presented as mean and standard deviation. ∆ angiogram-to-CMR, ∆ CMR I-to-CMRII are presented as median with interquartile range

*CMR* Cardiac magnetic resonance, *LV-EF* left ventricular ejection fraction, *LV-EDVI* left ventricular end diastolic volume index, *LGE* late gadolinium enhancement

*Wilcoxon matched-pairs signed rank test

### MACE and Clinical follow-up analysis

**Fig. 2:**
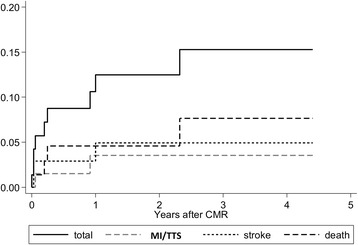
Kaplan-Meier graph of events showing the proportion of major adverse clinical events

All 72 patients were evaluated for follow-up after a median of 24 months (IQR 6–43). We observed 9 (12%) events in the study population of which four occurred in-hospital within 30 days after TTS. In-hospital mortality was 1%. Three patients died from non-cardiac events (malignancy) under the medication of dual or mono antiplatelet therapy. Long-term follow up of patients revealed an estimated mortality rate of 5% after two years and 8% after three years including deaths of any cause (Fig. 2 and Table 4). In two patients stroke occurred in the first 30 days with a left ventricular thrombus in one patient. None of these patients received prior oral anticoagulation, but antiplatelet therapy. Another patient had a stroke event twelve months after TTS under oral anticoagulation due to atrial fibrillation. One patient suffered from recurrent TTS twelve months after the first event under dual antiplatelet therapy, BB and ACEi. In one patient we observed myocardial infarction under antiplatelet monotherapy (Table 5).

**Table 4:** Description of MACE

Kaplan-Meier estimation			
		estimated rate (95%-confidence interval)	
MACE		24 months	36 months
Total events	9 (12%)	12.5 (6.4–23.5)	15.3 (8.0–28.0)
Death	4 (5.6%)	4.6 (1.5–13.5)	7.6 (2.8–20.3)
Stroke	3 (4.2%)	4.9 (1.6–14.7)	4.9 (1.6–14.7)
MI/TTS	2 (2.8%)	3.5 (0.9–13.6)	3.5 (0.9–13.6)

Data is presented as number of patients and percentage. Estimated event rates are presented with Kaplan-Meier estimation after 24 and 36 months

*MACE* major adverse clinical events, *MI* myocardial infarction, *TTS* Takotsubo syndrome

**Table 5:** Description of MACE depending on prior antithrombotic therapy

						Study population *n* = 72
MACE	No AT *n* = 3	AT *n* = 28	Dual AT *n* = 29	OAC *n* = 6	AT + OAC *n* = 4	Triple therapy *n* = 2
No event	2 (3%)	25 (35%)	25 (35%)	5 (7%)	4 (6%)	2 (3%)
With Event	1 (1%)	3 (4%)	4 (6%)	1 (1%)	0	0
Myocardial infarction	0	1 (1%)	0	0	0	0
Stroke	0	1 (1%)	1 (1%)	1 (1%)	0	0
Recurrence of TTS	0	0	1 (1%)	0	0	0
Death	1 (1%)	1 (1%)	2 (3%)	0	0	0

Data is presented as number of patients and percentage

*MACE* major adverse cardiac event, *AT* antiplatelet therapy, *OAC* anticoagulation therapy

## Discussion

To date this is the first study focusing on the evaluation of therapy management in relation to outcome after TTS. Main findings of our study were that patients with TTS receive antithrombotic therapy in most cases with a maximal duration of twelve months. Combination of antiplatelet therapy, oral anticoagulation and duration varied brightly. Dual antiplatelet therapy with a specific duration was highly recommended at discharge. Implementation rate was high and bleeding rate was low. Heart failure medication with BB and ACEi at discharge was found in the majority of patients. Left ventricular function and myocardial oedema improved after two months. MACE rate was moderate with an event rate of 12% and a 30-days mortality rate of 1%.

### Clinical findings and emotional triggers

Our clinical findings, such as the female emphasized distribution in TTS patients, the coexisting neurologic or psychiatric disorders and evaluation of emotional triggers is in line with previous studies [1, 18–21].

### Antiplatelet therapy

Previous studies indicate that blood hyperviscosity is a part of pathophysiology in TTS. Blood viscosity, erythrocyte membranes and endothelial integrity were altered in patients with previous TTS that were exposed to sympathetic stimulation matched to a control group with chest pain and without coronary artery disease [8]. A significant increase of endothelial dysfunction was found in another study on 22 TTS patients matched to a control group [22]. Interestingly, there was no difference in these patients regarding arterial stiffness and intima-media thickness. However, intracoronary thrombosis and plaque rupture were not observed in TTS by an intracoronary ultrasound study [9]. These findings underline that vascular and endothelial dysfunction plays a role in the pathophysiology of TTS and that patients might benefit from antiplatelet therapy. In our study we have found that the majority of patients received antiplatelet therapy after discharge. Recommendations were implemented in the majority of patients. Bleeding rate after antithrombotic therapy was low. The medication with antiplatelet therapy might have a beneficial effect. This might be one explanation why MACE rates and mortality in our study was relatively low compared to previous register data [1, 23]. In our subgroup analysis we observed slightly higher stroke rates within the first 30 days compared to previous reports. One explanation might be a potential formation of a left ventricular thrombus facilitated by apical ballooning and apical wall motion abnormalities. In our study, we observed one patient with left ventricular thrombus within the first 30 days after admission. Oral anticoagulation in the first 30 days might have a positive effect on decreasing stroke rates in TTS. Previous case series indicate that thrombus formation in TTS is rarely reported and that most thrombi were detected within the first 2 weeks, in some cases despite oral anticoagulation [24]. There is definitely a lack on large multicenter studies regarding the use and duration of antithrombotic medication, which needs further investigation to state a future guideline.

### Heart failure therapy and TTS recurrence

Available data on the effect of BB on acute heart failure of TTS are ambivalent. In previous studies patients with intraventricular pressure gradients improved from intravenous application of BB [25–27]. In a case series with left ventricular outflow tract (LVOT) obstruction in acute TTS treatment with BB decreased LVOT gradient within some days [28]. Some preclinical studies have shown a benefit in the use of BB [29, 30]. One possible explanation for the ineffectiveness of BB that is currently discussed is involvement of ß2-receptors in TTS, rather than ß1–adrenoceptor stimulation. However, a meta-analysis on the efficacy of medical treatment of TTS patients [26] did not reveal a positive effect of BB, ACEi/ARB and statins on the recurrence rate of TTS. In a large cohort of patients Templin et al. [1] revealed that the use of ACEi/ARB were associated with improved survival at 1 year. The authors discussed a reduction in sympathetic activity or anti-inflammatory effects of ACEi on myocardium that might explain the reduction in recurrence rate. However BB did not show any benefit at 1 year in the same study. Furthermore, use and prescription of antiplatelet therapy was not analyzed in that study. In our study 30-days mortality was also low. The majority of patients in our study were treated with BB and ACEi. If low mortality rate is caused by the medication with BB and ACEi medication or by antithrombotic medication or the combination of both has to be investigated in further studies.

To date there is no evidence for prevention of TTS recurrence. Effects of BB and ACEi/ARB on the prognosis of TTS patients still remain ambivalent. Overall, data on TTS recurrence is low, since cases described are rare. In our cohort we have found one patient with recurrent TTS. This patient was treated with BB and ACEi without recommendation of duration at discharge. The use of BB has been proposed to prevent TTS recurrence in some reports [25]. In a systematic review on incidence of TTS recurrence 31 cohorts (1664 TTS patients) were included [31]. Out of these 74 cases of recurrence after a mean follow-up of 24.5 months were described. Annual recurrence incidence was 1–2%. Discharge medications included BB in 66.8%, ACEi and ARB in 67.4%. Recurrence rate correlated with ACEi/ARB prescription was significantly lower. The recurrence rate in our study is in line with these data, however, further studies with larger cohorts need to prove this trend.

### MACE

We observed a 30-day mortality rate of 1% and an estimated mortality rate of 5% after two years including all causes of death. Prior data on long-term prognosis in TTS are ambivalent. Our analyses are in line with reports on a comparable mortality rate to an age- and gender matched population and to a large cohort study [1, 32]. One explanation for the low mortality rate in our study might be that the majority of patients were treated with antithrombotic and heart failure medication. Redfors et al. described higher mortality rates, which can be compared to NSTEMI and STEMI patients. However, only 30% of these patients were treated with dual antiplatelet therapy and 45% of patients received BB [23]. Stroke rate in our study was 4% after two years, which is higher than previously reported [1, 23]. Templin et al. described a stroke rate of 1.7% per patient-year [1]. In our study patients with acute stroke were treated with prior antiplatelet therapy. One hypothesis is that oral anticoagulation might have decreased these stroke events and should be considered until full recovery of left ventricular function. Currently, our investigation is the first study that has analyzed prognosis of TTS in regard to different antithrombotic therapy strategies.

### CMR

In our cohort mean LV-EF during index event was 49.1% ± 10.1. After two months we found a complete recovery of LV-EF and a significant reduction of myocardial oedema in the majority of TTS patients. These findings are similar to previous reports [1]. First CMR studies on left ventricular function indicated a recovery in most patients after 6 months [4]. Further studies observed a faster recovery in the majority of patients [33–35]. Our data confirm that left ventricular function in TTS recovers rapidly. We therefore assume that medication used to treat heart failure is less effective after the first two months of TTS.

### Study limitation

Due to the retrospective study design our analysis has following limitations: Not every patient was analyzed for a follow-up CMR, which compromises our CMR follow-up data. CMR was used as a major cardiac imaging tool for the diagnosis of TTS. Patients’ number of Takotsubo cases over the years might be underestimated due to patients that could not be investigated through CMR because of contraindications to CMR or cardiogenic shock. Median time between angiogram and CMR was two days, which is already a rapid interval. However, previous literature has reported quick changes of left ventricular function within days or weeks. The few amounts of patients with normal LV-EF or lack of typical wall motion abnormalities in the initial CMR scan might be explained due to the fact that an interval of two days might not be short enough to represent all courses of TTS. Also we excluded patients with prior myocardial infarction to distinguish clearly TTS patients for this study. Patients with TTS and coexisting coronary artery disease might have different results and need further evaluation in future studies. Although we have performed an analysis on a relatively high number of TTS patients gained during a long period of time we achieved small numbers in the subgroup analysis regarding different antithrombotic therapy strategies. Conclusions that are drawn from the subgroup analysis have to be interpreted carefully and therefore, prospective multicenter studies need to be conducted to confirm our findings.

## Conclusions

Currently, this is the first study analyzing prescribed therapy, therapy duration in TTS and prognosis depending on different therapy strategies. Although no therapy guidelines in TTS exist so far, the majority of patients in our study were treated with antithrombotic and heart failure therapy for a maximum of twelve months. Left ventricular function and myocardial oedema recovered rapidly within the first two months. Outcome analysis showed a low bleeding rate and a high short-term survival. Therefore, TTS patients might benefit from antithrombotic and heart failure therapy at least for the first two months. Further prospective studies need to be conducted to evaluate the optimal treatment strategy for TTS.

## Abbreviations

ACEi: Angiotensin converting enzyme receptor inhibitors
ARB: Angiotensin receptor blockers
ASA: Acetylsalicyl-acid
BB: ß-adrenoceptor antagonists
CK: Creatine-kinase
CK-MB: Creatine-kinase myocardial band
CMR: Cardiovascular magnetic resonance imaging
CRP: C-reactive protein
GFR: Glomerular filtration rate
LGE: Late gadolinium enhancement
LV-EF: Left ventricular ejection fraction
MACE: Major adverse clinical events
MRA: Mineralocorticoid receptor antagonists
OAC: Oral anticoagulation
TTS: Takotsubo-Syndrome
